# Application of Scaffold-Based Drug Delivery in Oral Cancer Treatment: A Novel Approach

**DOI:** 10.3390/pharmaceutics16060802

**Published:** 2024-06-14

**Authors:** Elham Saberian, Andrej Jenča, Adriána Petrášová, Hadi Zare-Zardini, Meysam Ebrahimifar

**Affiliations:** 1 Klinika and Akadémia Košice, Pavol Jozef Šafárik University, n.o. Bačíkova 7, 04001 Kosice, Slovakia; el_saberian@yahoo.com; 2 Klinika of Stomatology and Maxillofacial Surgery Akadémia Košice, UPJS LF, Pavol Jozef Šafárik University, n.o. Bačíkova 7, 04001 Kosice, Slovakia; andrej.jenca1@upjs.sk (A.J.); adriana.petrasova@upjs.sk (A.P.); 3 Department of Biomedical Engineering, Meybod University, Meybod 89616-99557, Iran; 4 Department of Toxicity, Faculty of Pharmacy, Islamic Azad University, Shahreza Branch, Shahreza 81796-35875, Iran

**Keywords:** scaffold, drug delivery, nanocarriers, oral cancer

## Abstract

This comprehensive review consolidates insights from two sources to emphasize the transformative impact of scaffold-based drug delivery systems in revolutionizing oral cancer therapy. By focusing on their core abilities to facilitate targeted and localized drug administration, these systems enhance therapeutic outcomes significantly. Scaffolds, notably those coated with anti-cancer agents such as cisplatin and paclitaxel, have proven effective in inhibiting oral cancer cell proliferation, establishing a promising avenue for site-specific drug delivery. The application of synthetic scaffolds, including Poly Ethylene Glycol (PEG) and poly(lactic-co-glycolic acid) (PLGA), and natural materials, like collagen or silk, in 3D systems has been pivotal for controlled release of therapeutic agents, executing diverse anti-cancer strategies. A key advancement in this field is the advent of smart scaffolds designed for sequential cancer therapy, which strive to refine drug delivery systems, minimizing surgical interventions, accentuating the significance of 3D scaffolds in oral cancer management. These systems, encompassing local drug-coated scaffolds and other scaffold-based platforms, hold the potential to transform oral cancer treatment through precise interventions, yielding improved patient outcomes. Local drug delivery via scaffolds can mitigate systemic side effects typically associated with chemotherapy, such as nausea, alopecia, infections, and gastrointestinal issues. Post-drug release, scaffolds foster a conducive environment for non-cancerous cell growth, adhering and proliferation, demonstrating restorative potential. Strategies for controlled and targeted drug delivery in oral cancer therapy span injectable self-assembling peptide hydrogels, nanocarriers, and dual drug-loaded nanofibrous scaffolds. These systems ensure prolonged release, synergistic effects, and tunable targeting, enhancing drug delivery efficiency while reducing systemic exposure. Smart scaffolds, capable of sequential drug release, transitioning to cell-friendly surfaces, and enabling combinatorial therapy, hold the promise to revolutionize treatment by delivering precise interventions and optimized outcomes. In essence, scaffold-based drug delivery systems, through their varied forms and functionalities, are reshaping oral cancer therapy. They target drug delivery efficiency, diminish side effects, and present avenues for personalization. Challenges like fabrication intricacy, biocompatibility, and scalability call for additional research. Nonetheless, the perspective on scaffold-based systems in oral cancer treatment is optimistic, as ongoing advancements aim to surmount current limitations and fully leverage their potential in cancer therapy.

## 1. Introduction 

Cancer encompasses a variety of diseases characterized by the uncontrolled proliferation and division of abnormal cells that can invade and damage surrounding tissues and organs. These malignancies can originate in various parts of the body, including the stomach, lungs, brain, and breasts [1,2,3,4,5,6,7,8,9]. Oral cavity cancer, a significant global health concern, manifests in areas such as the lips, hard palate, alveolar ridges, tongue, sublingual region, buccal mucosa, and posterior deltoid muscle of the molars [10,11,12,13,14,15]. It ranks among the ten most common malignancies worldwide, with approximately 34,000 new cases annually in the United States alone [16,17,18,19,20]. Oral squamous cell carcinoma (OSCC) is the predominant subtype, accounting for over 90% of oral cancer cases [21].

Despite advances in treatment options like surgery, chemotherapy, and radiotherapy, the mortality rate for OSCC remains high due to its propensity for lymphatic spread and metastasis [22,23,24,25,26,27,28]. Chemotherapy often results in severe side effects such as nausea, vomiting, hair loss, infections, and diarrhea, which can outweigh the benefits of treatment in non-aggressive cancer forms [29,30,31,32]. Surgical resection, while primary, often leads to permanent disfigurement, functional impairment, and reduced quality of life. Consequently, the five-year survival rate for patients remains between 50–60% due to late diagnosis and increased likelihood of local recurrence and distant metastasis [33,34,35,36,37,38].

To address these challenges, innovative approaches such as targeted therapies are being explored. These therapies allow for the local administration of drugs in higher concentrations, minimizing systemic toxicity and improving patient survival [39,40,41,42,43,44,45,46]. Currently, anti-cancer drugs like 5-fluorouracil, paclitaxel, cisplatin, and docetaxel are used in oral cancer treatment [47,48]. However, their intravenous administration can be highly toxic to healthy cells and limit their efficacy due to poor solubility, permeability, and bioavailability [49,50]. This underscores the urgent need for new therapeutic regimens or modifications to existing approaches to improve treatment outcomes and minimize damage to healthy tissues.

Scaffold-based drug delivery systems offer a promising solution to these limitations. Scaffolds, due to their biocompatibility and ability to act as carriers for targeted drug delivery, are particularly beneficial in oral cancer treatment. These systems enable localized delivery of therapeutics, reducing systemic side effects and improving therapeutic outcomes [51,52,53]. The choice of scaffold materials is critical, affecting drug loading, release duration, and efficacy against different cancer cell lines [47,48,49,50]. By providing a platform for the controlled release of chemotherapeutics, scaffold-based systems have the potential to transform oral cancer treatment through more precise and targeted therapeutic interventions, ultimately enhancing patient outcomes [51].

The scientific rationale for conducting this narrative review stems from the need to explore novel therapeutic approaches that can overcome the limitations of current treatment modalities. Traditional therapies, while effective to some extent, are often accompanied by significant adverse effects and limitations in efficacy, particularly in advanced stages of the disease. Therefore, there is a pressing need to identify and evaluate innovative strategies that can enhance treatment outcomes while minimizing side effects.

One such promising strategy is the use of scaffold-based drug delivery systems. These systems offer targeted or localized drug delivery, which reduces systemic side effects and improves therapeutic efficacy. By providing a controlled and sustained release of multiple drugs, scaffold-based systems can potentially revolutionize the treatment landscape for oral cancer.

The main objective of this review is to provide a comprehensive overview of scaffold-based drug delivery systems and their application in oral cancer treatment. Specifically, this review aims to:

1. Elucidate the mechanisms by which scaffold-based systems enhance drug delivery and effectiveness compared to conventional techniques
2. Highlight the advantages of scaffold-based drug delivery systems over other targeted delivery methods, such as nanoparticles and nanolipids
3. Discuss the current state of research and potential future directions for scaffold-based therapies in oral cancer treatment

## 2. The Role of Scaffold-Based Drug Delivery in Oral Cancer Treatment

For optimal therapeutic efficacy, it is crucial that the administered drug is targeted to the tumor site and then selectively absorbed by the cancer cells. This method improves therapeutic efficacy by increasing drug concentration in tumor tissue, thereby reducing systemic toxicity and maximizing anti-tumor response [54,55]. Local administration of a drug-releasing scaffold in various tumor types has been shown to facilitate the prolonged release of cytotoxic/immunomodulatory agents at specific tumor sites, thereby reducing systemic exposure and associated sequelae and minimizing the need for repeated chemotherapy cycles and the associated financial burden on patients [56,57]. Considering the different types and stages of cancer, novel drug delivery systems using injectable hydrogels or scaffolds have emerged as smart solutions. These advanced delivery systems are designed to respond to various stimuli such as light, temperature, pH, and multiple other triggers. By incorporating stimulatory properties, these hydrogels/scaffolds can facilitate the targeted release of drugs, increasing therapeutic efficacy while mitigating side effects. The development of these intelligent delivery systems has significantly improved treatment prospects for cancer patients [56,58]. Advances in cancer biology and the wide availability of various biomaterials have greatly influenced the development of nanotechnology and state-of-the-art scaffolds for drug delivery to tumor tissue. These innovative systems hold promise for improving treatment outcomes by efficiently targeting malignant cells while minimizing damage to healthy tissue [59,60,61,62,63,64]. Nanomedicines have made remarkable progress in cancer therapy, with pioneers such as Doxil^®^ (liposomal doxorubicin) and Abraxane^®^ (albumin-bound paclitaxel) leading the way. These first-generation nanomedicines have brought tangible benefits to cancer patients. More recently, research has shifted to nanoparticles such as polymeric nanoparticles and liposomes, which are used to create 3D scaffolds that can improve local and specific drug delivery. This targeted approach holds promise for improving therapeutic outcomes [65,66,67]. In malignancies such as cancer, rapid angiogenesis leads to an abnormal vascular structure characterized by twisted, dilated, and permeable blood vessels, resulting in the phenomenon known as enhanced permeability and retention (EPR) [68]. This effect contributes significantly to the efficacy of drug delivery to tumors, especially in nanoparticle-based treatments. It allows drugs to accumulate in tumor tissue, leading to better therapeutic outcomes. By harnessing the EPR effect, researchers can develop targeted nanoparticle-based drug delivery systems that selectively concentrate drugs in tumors, reduce systemic toxicity, and maximize anti-tumor activity [68,69,70]. Regulation of the EPR effect is primarily influenced by several factors, including drug surface properties, size and shape, targeting mechanisms, and circulation time. These factors have a direct or indirect effect on the distribution and fate of drugs in tumors. However, some limitations must be considered when using the EPR effect for drug delivery, such as differences in the tumor microenvironment and heterogeneity between different tumor types, models, and patients [70,71]. The tumor microenvironment plays an important role in the development and optimization of nanoparticles and their carriers. By incorporating scaffolds into nanoparticle design, researchers can create systems that produce synergistic anti-cancer effects by releasing multiple agents from a single carrier while addressing the challenges associated with the release of hydrophobic agents [72,73,74].

In addition, advances in nanotechnology have led to the development of nano-drug carriers such as nanoparticles that have the potential to reverse the immunosuppressive microenvironment of tumors. Studies have shown that these carriers can increase the efficacy of immunotherapy by improving the transport and targeting of therapeutic agents to cancer cells while minimizing systemic toxicity. By leveraging the unique properties of nanomaterials, nano drug carrier systems offer a promising approach to overcoming the challenges associated with conventional chemotherapy and improving anti-tumor response [75]. Nanoparticle-based drug delivery systems have attracted considerable attention in cancer therapy due to their ability to increase the efficacy of anti-cancer agents while minimizing side effects. Various approaches and materials, including polymers, lipids, organic and inorganic materials, and ceramics, are being extensively studied for their potential therapeutic benefits. Some of these materials have shown promising results in preclinical studies. The versatility of nanoparticles allows their physicochemical properties to be tailored to optimize drug delivery, targeting, and pharmacokinetics to improve treatment outcomes [76,77]. Harnessing the toxicity of drugs as antineoplastic agents at tumor sites has shown that they can eradicate malignant cells and effectively control tumors to prevent recurrence. However, these methods have also destroyed healthy cells in neighboring tissues, leading to systemic side effects [78].

Scaffolds, which act as a targeted or localized transport system for cytotoxic agents, are becoming increasingly important as a promising approach in the search for effective tumor therapy. Scaffolds have the potential to significantly improve the efficacy and safety of antineoplastic treatments by selectively transporting therapeutic agents to cancer cells while sparing healthy tissue [79]. These injectable scaffolds offer a less invasive method of drug delivery and allow for precise and regulated release of therapeutics. They can be engineered to deliver multiple drugs synchronously for a versatile therapeutic effect. This strategy has the potential to improve treatment outcomes and minimize side effects compared to traditional methods of drug delivery. In addition, these scaffolds can be tailored to provide structural support to damaged tissue, facilitating repair and regeneration. Overall, injectable scaffolds represent a promising way to treat various diseases [80]. Intratumoral delivery of cancer vaccines has shown potential in the treatment of various cancers. However, this approach presents a number of challenges. These include concerns regarding biodegradation, immune response, and ensuring efficient and targeted drug delivery. In addition, controlling the spatiotemporal release of cancer drugs from the depot can be challenging, and proper administration requires specialized training. While intratumoral delivery of vaccines shows promise, further research is essential to overcome these obstacles and optimize efficacy [81].

Scaffold-based drug delivery systems have emerged as a promising strategy in oral cancer therapy, as they allow for targeted or localized delivery of drugs that can mitigate systemic side effects and improve treatment efficacy [51,52,53]. In addition, the potential use of cancer drugs coated on scaffolds has been explored for local drug delivery in oral cancer therapy, with evidence of inhibition of cancer cell proliferation. In addition, scaffold-based drug delivery systems promise to improve treatment outcomes and minimize side effects in oral cancer therapy. The selection of scaffolds and chemotherapeutic agents depends on the specific cancers to be treated, and the development of scaffolds capable of regulating the rate of chemotherapy delivery to the target site is considered essential. Consequently, scaffold-based drug delivery systems represent a promising avenue for oral cancer therapy that requires further investigation to refine their efficacy and safety [53,82].

## 3. How Do Scaffold-Based Drug Delivery Systems Compare to Other Targeted Drug Delivery Systems in Oral Cancer Treatment?

Scaffold-based drug delivery systems have proven to be a promising method in oral cancer therapy. They allow for targeted or localized drug delivery, which can reduce systemic side effects and increase treatment efficacy. Compared to other targeted drug delivery systems such as nanoparticles and nanolipids, scaffold-based drug delivery systems can deliver drugs in a minimally invasive manner or implant them in situ. They can also release multiple drugs in a controlled and sustained manner, resulting in a versatile therapeutic effect [51,53]. In addition, scaffold-based drug delivery systems can mitigate the severity and extent of side effects, providing a new therapeutic approach for patients with oral, head and neck cancer and beyond [53]. In addition, the potential use of anti-cancer drug-coated scaffolds for local drug delivery in the treatment of oral cavity cancer has been explored, with inhibition of cancer cell proliferation observed [83]. Overall, scaffold-based drug delivery systems offer numerous advantages over other targeted drug delivery systems. These include targeted or localized delivery of therapies, reduced systemic side effects, and improved treatment outcomes.

One of the key mechanisms by which scaffold-based systems enhance medication delivery is through their structural design. Scaffolds are often made from biocompatible and biodegradable materials, which can be engineered to have specific pore sizes and surface properties. These features allow scaffolds to encapsulate and protect the drug molecules, ensuring a steady and controlled release over time. This sustained release profile helps maintain therapeutic drug concentrations at the target site, thereby improving the efficacy of the treatment and reducing the frequency of administration [51,52,53,54,55].

Additionally, scaffolds can be designed to release drugs in response to specific physiological triggers, such as changes in pH or temperature, which are often associated with the tumor microenvironment. This targeted release mechanism ensures that the drug is delivered precisely where and when it is needed, minimizing exposure to healthy tissues and reducing systemic toxicity [84].

Scaffold-based systems also enhance drug delivery through their ability to provide a supportive microenvironment for cell growth and tissue regeneration. This is particularly beneficial in oral cancer treatment, where tissue damage from surgery or radiation therapy can impede healing. By promoting tissue regeneration, scaffolds help restore the normal function of the affected area, aiding in the overall recovery process [80,85,86].

Moreover, the incorporation of bioactive molecules, such as growth factors or signaling peptides, into the scaffold matrix can further enhance the therapeutic effect. These molecules can stimulate the immune response, inhibit tumor growth, or sensitize cancer cells to chemotherapy, providing a multifaceted approach to cancer treatment.

The use of scaffold-based drug delivery systems in oral cancer treatment has shown promising results in preclinical studies. For instance, anti-cancer drug-coated scaffolds have demonstrated the ability to inhibit cancer cell proliferation effectively. This localized drug delivery approach not only targets the tumor more efficiently but also helps in mitigating the severity and extent of side effects commonly associated with conventional therapies [86,87].

In summary, scaffold-based drug delivery systems offer several advantages over other targeted drug delivery methods, including:

- Targeted or localized delivery: ensures high drug concentration at the tumor site while minimizing systemic exposure
- Controlled and sustained release: maintains therapeutic drug levels over extended periods, reducing the need for frequent dosing
- Responsive release mechanisms: delivers drugs in response to specific tumor-related triggers, enhancing precision
- Supportive microenvironment: promotes tissue regeneration and overall healing in the affected area
- Multifaceted therapeutic approach: incorporates bioactive molecules to enhance the overall therapeutic effect

These mechanisms collectively contribute to the enhanced medication delivery and effectiveness of scaffold-based systems, making them a superior option for treating oral cancer compared to conventional techniques.

Table 1 showed the comparative data on treatment outcomes: scaffold-based systems vs. conventional therapies. 

## 4. Different Types of Scaffold-Based Drug Delivery in Oral Cancer Treatment

In the management of oral cancer, researchers have investigated a range of drug delivery systems based on scaffolds (Table 2). 

These systems offer the potential for targeted or localized drug delivery, reducing systemic side effects and improving therapeutic outcomes. The mechanisms of drug release from scaffold-based systems are summarized in Table 3. 

### 4.1. Three-Dimensional (3D) Scaffolds

Scaffold-based drug delivery systems, especially three-dimensional (3D) scaffolds, have proven to be a promising approach for the treatment of oral cancer. These scaffolds can serve as implantable or injectable delivery platforms for anti-tumor agents. They provide a minimally invasive method of drug delivery and allow for the controlled and sustained release of multiple drugs, resulting in a multifunctional therapeutic effect [51,84]. The 3D scaffolds have also been shown to induce anti-tumor immunity, promote cell expansion, and improve anti-tumor efficiency, making them a promising strategy for cancer treatment [84]. Moreover, these scaffolds can respond to biological signals, adjust their properties accordingly, and enable targeted, sustained, and controlled delivery of chemotherapeutic agents, thereby removing the obstacles of drug resistance and improving therapeutic outcomes [51]. In addition, 3D scaffolds have been shown to induce tumoroids, providing a platform for studying the mechanisms of tumorigenesis and evaluating anti-cancer drugs [85]. The development of smart scaffolds for sequential cancer therapy has been proposed to improve the efficacy of drug delivery systems and minimize the need for multiple surgical interventions [80].

Three-dimensional (3D) scaffolds play a central role in the treatment of oral cavity cancer. They allow for targeted or localized delivery of therapeutics, thereby reducing systemic side effects and improving therapeutic outcomes [51,85,86]. These scaffolds have been used extensively for the engineering of various oral tissues. They provide robust support for cell development and mimic various mechanical and biochemical properties [86]. In oral cancer research, 3D scaffolds have proven to be valuable tools for studying the mechanisms of tumorigenesis and evaluating anti-cancer drugs [85]. They enable the production of tumoroids from tumor cell lines and biopsies and allow for the cultivation of tumor biopsies from patients to evaluate the efficacy of anti-cancer drugs. In addition, 3D scaffolds have been proposed as a platform for the development of personalized cancer therapies tailored to the individual needs of patients [85]. In addition to their role in drug delivery, 3D scaffolds have also attracted interest for their potential in cancer immunotherapy. Injectable or implantable hydrogels and scaffolds, representing macroscale 3D biomaterials, enable the controlled delivery and release of therapeutic agents that modulate the behavior of immune cells and increase the efficacy of immunotherapies [87]. Various types of 3D scaffolds are used in the treatment of oral cancer, including:

- Natural Scaffolds

Natural scaffolds have been investigated in oral cancer research as potential components of targeted drug delivery systems. These scaffolds can take the form of hydrogels or nanocarriers such as nanoparticles and nanolipids and represent a promising way to deliver anti-cancer agents locally to the tumor, thereby reducing systemic side effects and improving therapeutic outcomes [51,88]. Scaffolds made from natural materials, such as collagen, Matrigel, or silk, or from synthetic materials, such as polyethylene glycol (PEG) or poly(lactic-co-glycolic acid) (PLGA), or a combination of both have been used for 3D scaffold-based drug delivery systems [86,88]. Injectable or implantable hydrogels and scaffolds, which are macroscale 3D biomaterials, facilitate the controlled delivery and release of therapeutic agents, modulate the behavior of immune cells, and increase the efficacy of immunotherapies [87]. There is a proposal for the development of a smart scaffold for sequential cancer therapy to improve the efficiency of drug delivery systems and minimize the need for multiple surgical interventions [87].

- Synthetic Scaffolds

In oral cancer research, synthetic scaffolds such as polyethylene glycol (PEG) and poly(lactic-co-glycolic acid) (PLGA) have been investigated for their utility in 3D scaffold-based drug delivery systems. These synthetic scaffolds can effectively mimic the physiological environment and enable the production and controlled release of therapeutic agents, facilitating various anti-cancer interventions [51,89]. In addition, synthetic scaffolds provide robust support for cell growth and can mimic various mechanical and biochemical properties, making them a promising platform for the study of oral cancer and the development of targeted drug delivery systems [51]. In addition, there is a proposal for the development of a smart scaffold for sequential cancer therapy, which aims to improve the efficacy of drug delivery systems and reduce the need for multiple surgical procedures. This highlights the potential of synthetic scaffolds to advance the treatment of oral cancer [80].

- Scaffold-Free Strategies/Scaffold-Based Strategies

In oral cancer research, the use of three-dimensional (3D) scaffolds for drug delivery has aroused great interest. These scaffolds can be used in both scaffold-based and scaffold-free strategies. In scaffold-based strategies, 3D scaffolds, such as injectable or implantable hydrogels, are used to facilitate the controlled delivery and release of therapeutic agents. This approach offers a promising route for local delivery of anti-cancer drugs to the tumor, reducing systemic side effects and improving therapeutic outcomes [51,87]. Conversely, scaffold-free strategies, such as cell sheet and spheroid approaches, do not rely on a scaffold for cell growth and organization. These strategies have been used to replicate the oral cancer microenvironment and to investigate the role of the immune system in oral cancer [86]. There is a proposal for the development of a smart scaffold for sequential cancer therapy to improve the efficacy of drug delivery systems and minimize the need for multiple surgical procedures. This highlights the potential of 3D scaffolds to advance the treatment of oral cancer [80]. Both scaffold-based and scaffold-free strategies using 3D scaffolds hold promise to revolutionize the treatment of oral cancer by enabling more precise and targeted therapeutic interventions and ultimately improving patient outcomes.

### 4.2. Anti-Cancer Drugs Coated Scaffolds

Scaffolds coated with cancer drugs such as cisplatin and paclitaxel have been shown to inhibit the growth of oral cancer cells, providing a potential route for localized drug delivery [82]. Synthetic scaffolds such as polyethylene glycol (PEG) and poly(lactic-co-glycolic acid) (PLGA) have been investigated for their role in 3D scaffold-based drug delivery systems that enable the production and controlled release of therapeutic agents, thereby facilitating various anti-cancer activities [90]. In addition, natural scaffolds, including collagen, Matrigel, and silk, have also been investigated for their potential in targeted drug delivery systems [90,91]. There is a proposal for the development of a smart scaffold for sequential cancer therapy, which aims to improve the efficacy of drug delivery systems and minimize the need for multiple surgical procedures, highlighting the potential of 3D scaffolds in the treatment of oral cancer [53]. Thus, cancer drug-coated scaffolds and other scaffold-based drug delivery systems promise to revolutionize the treatment of oral cancer by enabling more precise and targeted therapeutic interventions, ultimately improving patient outcomes.

The potential side effects of using scaffolds coated with anti-cancer drugs for the treatment of oral cancer are currently under investigation. However, the existing literature suggests that local delivery of anti-cancer drugs using these scaffolds may help to mitigate the systemic side effects associated with conventional systemic drug delivery [82]. Conventional systemic drug delivery can lead to various adverse effects, such as nausea, vomiting, hair loss, infections, and diarrhea [53]. In contrast, the use of drug-eluting scaffolds may help to minimize these systemic side effects by delivering the drugs directly to the tumor site, thereby reducing their effects on healthy tissues and cells [82]. Nevertheless, further research is needed to thoroughly evaluate the potential side effects of this approach.

The use of anti-cancer drug-loaded scaffolds in oral cancer therapy has been shown to impact the local environment by increasing drug bioavailability at the local site and reducing systemic side effects of anti-cancer drugs [82]. These drug-loaded scaffolds can be placed at the surgical site, and the gradual and sustained local release of cytotoxic drugs from the implanted scaffolds over a prolonged period of time can prevent the toxic side effects associated with systemic drug delivery [80]. In addition, once the drug is released, the scaffolds are non-toxic and promote cell growth, allowing non-cancerous cells to adhere and proliferate, making them a potential solution for an effective drug-carrying scaffold for volume replenishment [80]. The use of scaffolds as targeted or localized, toxicity-inducing implantable/injectable delivery platforms for anti-tumor agents is emerging as a promising approach that demonstrates the ability for controlled release of multiple agents.

The most common types of scaffolds used for drug delivery in the treatment of oral cancer are as follows:

- Polymeric scaffolds: polymeric scaffolds, including polyethylene glycol (PEG) and poly(lactic-co-glycolic acid) (PLGA), have been extensively studied for their suitability for 3D scaffold-based drug delivery systems [52,53].
- Natural scaffolds: natural materials such as collagen, Matrigel, and silk have been investigated for their potential for targeted drug delivery systems [52].
- Nanocarriers: scaffolds can be formulated as nanocarriers containing nanoparticles and nanolipids to enable the regulated delivery and release of therapeutic agents [52].
- Hydrogels: hydrogels are another common form of scaffolds used for drug delivery. They offer a promising approach for the local delivery of anti-cancer drugs to the tumor [52,53].

These scaffolds are designed to improve the availability of drugs at the specific site, reduce systemic side effects, and enable a uniform and controlled release of therapeutic agents, ultimately increasing the efficacy of oral cancer treatment [52,53].

### 4.3. Injectable Self-Assembling Peptide Scaffold Hydrogels

The use of injectable, self-assembling peptide scaffold hydrogels for the prolonged and controlled release of human antibodies offers a targeted approach to drug delivery for oral cancer therapy [92]. These self-assembling peptide hydrogels have been extensively studied for their potential for 3D scaffold-based drug delivery systems, providing a versatile and efficient platform for localized drug release. They provide a versatile and efficient platform for localized drug delivery. These hydrogels can be customized to provide sustained and controlled release of drugs, thereby improving drug availability at the target site and reducing systemic side effects commonly associated with conventional systemic drug delivery [93]. In addition, self-assembling peptides have been used as nano-cargos for targeted chemotherapy and immunotherapy of tumors and have the potential to improve the efficacy of cancer drug delivery systems [94]. This approach is promising for improving the efficacy of oral cancer therapy as it provides a more precise and targeted drug delivery system.

### 4.4. Dual Drug-Loaded Nanofibrous Scaffolds

Research has investigated the use of nanofiber scaffolds loaded with two drugs for potential postoperative cancer treatment and demonstrated the potential of this approach for oral cancer therapy [95]. These scaffolds, such as dual drug-loaded polyhydroxybutyric acid/gelatin nanofibers, have attracted much attention for the treatment of tissue defects after surgery for cancer [95]. The mechanism involves the sustained release of two drugs from the nanofibers, which can provide a synergistic therapeutic effect against cancer through the administration of multiple drugs [51]. The dual-drug-loaded nanofibrous scaffolds have the potential for targeted drug delivery by providing sustained and controlled release of therapeutic agents over a prolonged period of time, improving the availability of the drug at the site of action while minimizing systemic side effects [95]. The gradual and prolonged local release of cytotoxic drugs from the implanted scaffolds may prevent the adverse effects associated with systemic drugs.

### 4.5. Smart Scaffolds 

The development of smart scaffolds made of polymers has been proposed for sequential cancer therapy to improve the efficacy of drug delivery systems [80]. These smart scaffolds are designed to release drugs gradually and continuously and then transition to a surface that promotes cell growth [80]. The mechanism works according to the following scheme:

- Release of medication: smart scaffolds are designed to release drugs gradually and evenly, ensuring direct delivery of therapeutic agents to the affected region. This reduces the risk of side effects and increases the effectiveness of the treatment [80].
- Cell-friendly surface: once the drug is released, the scaffolds become non-toxic and cell-friendly, allowing non-cancerous cells to adhere and proliferate [80]. This property is particularly important for the treatment of oral cavity cancer, as it helps to fill the volume left by the removed tumor and promote tissue regeneration.
- Sequential therapy: The smart scaffolds can be designed to release multiple drugs or therapeutic agents simultaneously. This enables the development of combination therapies that can improve the efficacy of oral cancer treatment [53].
- Controlled degradation: The scaffolds can be structured with time-dependent degradation profiles that allow the controlled distribution of drugs over an extended period of time [53]. This property is crucial to maintain the therapeutic effect while reducing the potential for side effects.

The development of smart scaffolds for sequential cancer therapy holds great promise in revolutionizing therapeutic interventions by providing more precise and targeted therapeutic interventions, ultimately improving patient outcomes (Table 4).

**Figure 1:**
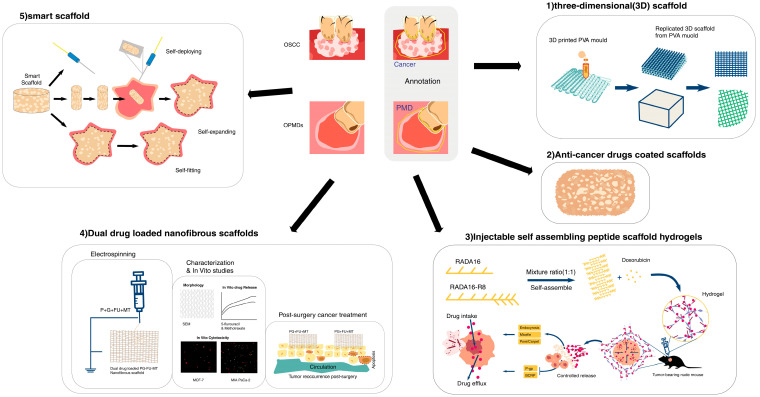
Types of scaffold-based drug delivery systems in oral cancer treatment.

In Figure 1, we summarized types of scaffold-based drug delivery systems in oral cancer treatment.

## 5. Future Outlook and Conclusion

The oral cavity holds immense significance for humans, with countless endeavors dedicated to preserving its well-being and addressing related issues [96,97,98,99,100]. In the treatment of oral cavity cancer, various scaffold-based drug delivery systems have been explored that offer the potential for targeted or localized drug delivery, thereby reducing systemic side effects and improving therapeutic outcomes. These systems include three-dimensional (3D) scaffolds that can be used as implantable or injectable delivery platforms for anti-tumor agents, allowing for the controlled and sustained release of multiple drugs, resulting in a multifunctional therapeutic effect.

Scaffolds coated with anti-cancer drugs such as cisplatin and paclitaxel have been shown to inhibit the proliferation of oral cancer cells, providing a potential approach for local drug delivery. Additionally, injectable self-assembling peptide scaffold hydrogels have been developed for the prolonged release of human antibodies, offering a targeted approach for drug delivery in oral cancer therapy. Furthermore, nanofiber scaffolds loaded with two drugs have been explored for potential postoperative cancer treatment, demonstrating the feasibility of this method in the treatment of oral cancer. The concept of polymer smart scaffolds for sequential cancer therapy has also been proposed to improve the efficiency of drug delivery systems.

These scaffold-based drug delivery systems offer promising opportunities for the treatment of oral cavity cancer with the potential for improved therapeutic outcomes and minimized side effects. However, several obstacles and limitations need to be addressed to fully realize their potential.

One major limitation is the complexity of fabricating scaffolds with precise control over their structural and functional properties. Achieving the desired drug release profile requires meticulous design and optimization, which can be technically challenging and time-consuming. Additionally, the biocompatibility and biodegradability of scaffold materials must be carefully evaluated to avoid adverse reactions in patients.

Another significant challenge is the potential for scaffold degradation, which can affect the stability and efficacy of the drug delivery system. Ensuring that the scaffold maintains its integrity and functionality over the required treatment period is crucial for effective therapy. Furthermore, the scalability of production and the cost-effectiveness of scaffold-based systems need to be considered to make these treatments accessible to a broader patient population.

The future prospects for scaffold-based drug delivery systems in oral cancer treatment are optimistic. Ongoing research is aimed at extending the duration of drug release, overcoming the challenges associated with scaffold-based cancer therapy, and developing smart scaffolds with customized structures and properties. These advances have the potential to further improve the efficacy and safety of oral cancer therapy and make scaffold-based drug delivery systems a key area for future advancements in cancer treatment.

In conclusion, while scaffold-based drug delivery systems present numerous advantages, including targeted drug delivery, reduced systemic side effects, and improved therapeutic outcomes, addressing the associated obstacles and limitations is crucial. Continued innovation and research in this field are essential to overcome these challenges and harness the full potential of scaffold-based therapies for the effective treatment of oral cavity cancer.

## Figures and Tables

**Table 1:** Comparative Data on Treatment Outcomes: Scaffold-Based Systems vs. Conventional Therapies.

Treatment Method	Drug Delivery System	Key Outcomes	Advantages	Limitations
Conventional Chemotherapy	Intravenous	High systemic toxicity, non-specific	Established method, rapid distribution	Severe side effects, non-targeted
Nanoparticle-Based Delivery	Nanoparticles	Improved targeting, reduced toxicity	Enhanced EPR effect, customizable	Complex fabrication, potential toxicity
Scaffold-Based Delivery	3D Scaffolds	Sustained release, targeted delivery	Reduced side effects, multifunctional	Manufacturing complexity, potential immune response
Injectable Hydrogels	Self-assembling peptides	Prolonged drug release, localized delivery	Minimally invasive, biocompatible	Stability issues, potential for uneven drug distribution
Drug-Coated Scaffolds	Coated with anti-cancer drugs	Inhibition of cancer cell proliferation	Localized delivery, reduced systemic exposure	Risk of scaffold degradation, need for precise engineering

**Table 2:** Types of Scaffold Materials Used in Oral Cancer Treatment.

Scaffold Material	Type (Natural/ Synthetic)	Key Properties	Applications in Oral Cancer Treatment
Collagen	Natural	Biocompatible, promotes cell adhesion	Used for creating 3D scaffolds to support tissue regeneration and drug delivery
Matrigel	Natural	Rich in ECM proteins, supports cell growth	Applied in tumor models for drug testing and delivery
Silk	Natural	High tensile strength, biocompatible	Used in scaffolds for controlled drug release and tissue engineering
Polyethylene Glycol (PEG)	Synthetic	Hydrophilic, biocompatible, tunable degradation rate	Used in hydrogels for sustained drug release and provides robust support for cell growth
Poly(lactic-co-glycolic acid) (PLGA)	Synthetic	Biodegradable, controllable degradation rate	Utilized in nanoparticles for targeted drug delivery, mimics physiological environment
Chitosan	Natural	Biodegradable, antimicrobial properties	Used for creating injectable hydrogels for local drug delivery

**Table 3:** Mechanisms of Drug Release from Scaffold-Based Systems.

Mechanism of Drug Release	Description	Advantages
Diffusion-Controlled Release	Drug diffuses out of the scaffold matrix over time	Provides sustained release, reduces dosing frequency
pH-Triggered Release	Drug release is triggered by changes in pH (e.g., acidic tumor microenvironment)	Enhances specificity, minimizes systemic toxicity
Temperature-Triggered Release	Drug release occurs in response to temperature changes	Allows for controlled release in response to body temperature
Enzyme-Triggered Release	Drug release is initiated by specific enzymes present in the tumor microenvironment	Targeted delivery, reduces off-target effects
Self-Assembling Peptide Hydrogels	Peptides self-assemble into hydrogels that release drugs over time	Prolonged release, biocompatible, injectable form

**Table 4:** Types and Applications of Scaffold-Based Drug Delivery Systems in Oral Cancer Treatment.

Scaffold Type	Key Properties	Applications in Oral Cancer Treatment
Natural Scaffolds	Biocompatible, supports cell growth	Targeted drug delivery, tumor modeling, tissue regeneration
Synthetic Scaffolds	Tunable properties, robust support for cells	Controlled drug release, mimicking physiological environment, sequential therapy
Anti-Cancer Drug-Coated Scaffolds	Localized drug delivery, inhibition of cancer cell growth	Localized chemotherapy, reduced systemic side effects
Injectable Self-Assembling Peptide Hydrogels	Prolonged drug release, customizable	Localized and sustained drug delivery, improved drug availability
Dual Drug-Loaded Nanofibrous Scaffolds	Sustained release of multiple drugs, synergistic effects	Postoperative cancer treatment, targeted therapy
Smart Scaffolds	Sequential drug release, cell-friendly surfaces	Combination therapies, tissue regeneration, controlled degradation

## Data Availability

No new data were created or analyzed in this study. Data sharing is not applicable to this article.
